# Sistemas de vigilancia epidemiológica en el medio laboral: una perspectiva en países de Latinoamérica

**DOI:** 10.15446/rsap.V25n4.99325

**Published:** 2023-07-01

**Authors:** Martín Gracia-Socha, Juan C. Cerón-Arcos, Alejandro Nocua-Salazar, Juan J. Vaca-González

**Affiliations:** 1 MG: MD. M. Sc. Salud Pública y Desarrollo Social. Esp. Gerencia en Seguridad y Salud en el Trabajo. Esp. Auditoría en Salud. Fundación Universitaria del Área Andina. Bogotá, Colombia. mgracias@unal.edu.co Universitaria del Área Andina Gerencia en Seguridad y Salud en el Trabajo Fundación Universitaria del Área Andina Bogotá Colombia mgracias@unal.edu.co; 2 JC: Enf. M. Sc. Salud Pública y Desarrollo Social. Esp. Gerencia en Seguridad y Salud en el Trabajo. Fundación Universitaria del Área Andina. Bogotá, Colombia. jcerona@areandina.edu.co Fundación Universitaria del Área Andina Gerencia en Seguridad y Salud en el Trabajo Fundación Universitaria del Área Andina Bogotá Colombia jcerona@areandina.edu.co; 3 AN: Opt. M. Sc. Salud Pública y Desarrollo Social. Esp. Gerencia en Seguridad y Salud en el Trabajo. Fundación Universitaria del Área Andina. Bogotá, Colombia. anocuas@areandina.edu.co Fundación Universitaria del Área Andina Fundación Universitaria del Área Andina Bogotá Colombia anocuas@areandina.edu.co; 4 JV: Ing. Automatización. M. Sc. Ingeniería Biomédica. Ph. D. Tecnología para la Salud y el Bienestar. Universidad Nacional de Colombia, Sede de La Paz. La Paz, Colombia. jjvacag@unal.edu.co Universidad Nacional de Colombia Universidad Nacional de Colombia Sede de La Paz La Paz Colombia jjvacag@unal.edu.co

**Keywords:** Monitoreo epidemiológico, servicios de vigilancia epidemiológica, salud laboral, condiciones de trabajo, América Latina *(fuente: DeCS, BIREME)*, Epidemiological monitoring, epidemiologic surveillance services, occupational health, working conditions, Latin America *(source: MeSH, NLM)*

## Abstract

A través de una revisión de artículos científicos se identificó información sobre la implementación de sistemas de vigilancia epidemiológica (SVE) gestionados por empleadores en el marco de sistemas de gestión en seguridad y salud en el trabajo (SG-SST) en Colombia y Latinoamérica entre 2010 y 2021. Se usaron las bases de datos PubMed, Science Direct, SciELO, Scopus, Dialnet y Gale para identificar investigaciones que utilizaron datos de SVE implementados por empleadores. Se describieron los temas tratados, los sectores económicos, las fechas de publicación, los países de origen, las poblaciones estudiadas, el alcance y los parámetros de calidad. Se evidenció que solo tres estudios usaron información de SVE derivada de SG-SST para vigilar la exposición de trabajadores a riesgos epidemiológicos y prevenir enfermedades laborales y accidentes. De los 24 estudios examinados, 21 no usaban información de SVE, siendo considerados hallazgos negativos. La falta de adherencia a las normas, la carencia de SVE, el desconocimiento de diferentes instancias de SVE y la ausencia de estudios adecuados resultan en una cobertura deficiente de los trabajadores expuestos a riesgos laborales. La mayoría de los estudios resaltan la necesidad de SVE en el ámbito laboral. También destacan la importancia de generar datos masivos, sistemáticos, confiables y comparables desde fuentes primarias como empresas y unidades productivas para apoyar las políticas públicas que garanticen la seguridad social y la salud de los trabajadores. Ante esta realidad y los escasos estudios citados, se concluye que no se conoce ni se puede sustentar adecuadamente la situación de los trabajadores en la región.

## Abstract (en)

Through a review of scientific articles, information was identified on the implementation of Epidemiological Surveillance Systems (EVS) managed by employers within the framework of Occupational Health and Safety Management Systems (SG-SST) in Colombia and Latin America between 2010. and 2021. PubMed, Science Direct, SciELO, Scopus, Dialnet, and Gale databases were used to identify research that used employer-implemented EVS data. The topics covered, economic sectors, publication dates, countries of origin, populations studied, scope and quality parameters were described. It was evident that only 3 studies used EVS information derived from SG-SST to monitor workers' exposure to epidemiological risks and prevent occupational diseases and accidents. Of the 24 studies examined, 21 did not use EVS information, being considered negative findings. The lack of adherence to standards, the lack of EVS, the lack of knowledge of different instances of EVS and the absence of adequate studies result in poor coverage of workers exposed to occupational risks. Most studies highlight the need for EVS in the workplace. They also highlight the importance of generating massive, systematic, reliable and comparable data from primary sources such as companies and productive units to support public policies that guarantee the social security and health of workers. Given this reality and the few studies cited, it is concluded that the situation of workers in the region is neither known nor adequately supported.

La seguridad y salud en el trabajo (SST) es un componente fundamental de la salud pública, no solo como elemento conceptual y normativo, establecido en Colombia por la Ley 100, sino también como una disciplina complementaria que contribuye significativamente al bienestar de la población 1. Según la Organización Panamericana de la Salud (OPS), la salud pública es la respuesta organizada de la sociedad para promover, mantener y proteger la salud comunitaria, así como prevenir enfermedades, lesiones e incapacidades 2. La Organización Internacional del Trabajo (OIT) resalta que las intervenciones en salud pública han evolucionado, incorporando no solo servicios clínicos, sino también intervenciones sociales relacionadas con la producción, el trabajo y el ambiente, entre otros aspectos 3. Esto subraya la importancia de la integración entre salud, trabajo y ambiente, ya que el desarrollo comunitario depende de la actividad laboral y el estado de salud de sus miembros, que pueden verse afectados por las condiciones laborales.

La vigilancia epidemiológica, definida por el Centro de Control y Prevención de Enfermedades (CDC) como la recolección, el análisis y la interpretación sistemática de datos de salud, es esencial para planificar, implementar y evaluar políticas de salud pública 4. Un SVE tiene como objetivo minimizar los efectos negativos de los riesgos ocupacionales y mejorar las condiciones de salud asociadas a los puestos de trabajo, las zonas de trabajo y los elementos del medio físico o natural involucrados en el proceso productivo 5,6. Los SVE permiten identificar factores de riesgo nocivos y peligrosos que pueden afectar la salud de los trabajadores y ocasionar enfermedades relacionadas con su ocupación.

La implementación de programas de SVE dentro de los sistemas de gestión en seguridad y salud en el trabajo (SG-SST) es crucial para obtener la información necesaria que sustente la toma de decisiones en salud laboral y que, desde el ámbito laboral, influya en la formulación de políticas públicas 7. La falta de información precisa y oportuna representa un problema significativo para los especialistas, los directivos y los funcionarios responsables de la salud de los trabajadores, tanto en el sector público como en el privado. Es esencial determinar si la información epidemiológica se utiliza efectivamente como insumo para la toma de decisiones y la gestión preventiva de la salud en el entorno laboral.

Históricamente, los sistemas de salud en Latinoamérica han enfrentado desafíos significativos para abordar los problemas contemporáneos derivados de epidemias, conflictos internos, cambio climático y diversos factores asociados con el contexto físico y social, como la inequidad en el acceso a modelos de atención centrados en las personas y las comunidades, que son componentes clave de la protección social 8. En Colombia, el Sistema General de Seguridad Social en Salud (SGSSS) establece un esquema en el que los empleados contribuyen al financiamiento del sector subsidiado, con un gasto en salud que representaba el 7,2% del producto interno bruto (PIB) en 2017, inferior al promedio de 8,8% de los países de la Organización para la Cooperación y el Desarrollo Económico (OCDE) 9,10. Este contexto subraya la importancia de regular los gastos en salud y realizar una identificación adecuada del origen de los eventos de salud, ya sean comunes o laborales.

En el marco de las reformas estructurales de los años noventa, en medio de crisis económicas y el auge del desempleo y la informalidad, se creó el Sistema General de Riesgos Profesionales (SGRP) como parte del SGSSS. Este sistema agrupa a entidades públicas y privadas, como el Ministerio de Trabajo, el Ministerio de Salud, la Protección Social, el Consejo Nacional de Riesgos Laborales y el Comité Nacional de Salud Ocupacional, con normas y procedimientos destinados a prevenir, proteger y atender a los trabajadores frente a enfermedades y accidentes laborales 11. Sin embargo, hasta la fecha no se ha logrado una cobertura efectiva en materia de riesgos laborales para todos los sectores, especialmente los trabajadores informales e independientes.

La vigilancia epidemiológica es una estrategia esencial para la detección temprana de enfermedades y proporciona datos cruciales para el análisis y el diseño de estrategias de salud pública. Además, permite la planificación de recursos necesarios para mejorar el estado de salud de la población 12. En Colombia, el Decreto 1443 de 2014, que reglamenta el SG-SST, define la vigilancia epidemiológica como la recopilación, el análisis y la interpretación sistemática de datos para la prevención de riesgos laborales 13. Este decreto establece que la vigilancia es indispensable para la planificación, la ejecución y la evaluación de programas de seguridad e higiene industrial, el control de trastornos y lesiones relacionadas con el trabajo, y la protección y promoción de la salud de los trabajadores. Además, se señala que los empleadores deben desarrollar acciones de vigilancia y construir indicadores pertinentes y adecuados 14.

La necesidad de obtener datos de SVE en Colombia y Latinoamérica es evidente; de hecho, la implementación de SG-SST, obligatoria desde 2014, es esencial para proteger a los trabajadores y las empresas frente a diversos riesgos laborales, incluidos los riesgos biológicos, físicos, químicos, mecánicos, ambientales y psicosociales 15. El desempleo, la informalidad y el subempleo están asociados a problemáticas que impactan significativamente la salud pública. Las condiciones laborales inadecuadas crean un círculo vicioso que afecta el bienestar, la salud individual y la calidad de vida de las personas 16. Por ello, es crucial contar con información derivada de la implementación de SVE que, en términos de pertinencia y oportunidad, permita entender la situación de salud en los distintos sectores laborales y aplicar mejoras en el marco general de la salud pública.

Existen múltiples obstáculos para establecer SVE de calidad en seguridad y salud en el trabajo. La mayoría de las empresas carecen de profesionales de la salud y de personal especializado en medicina del trabajo, epidemiología o sistemas de gestión, lo que resulta en la ignorancia o el subregistro de la morbilidad y la mortalidad en el entorno laboral 17. Además, la falta de una cultura de prevención en salud en los medios laborales y el desconocimiento de la normatividad por parte de los trabajadores comunes contribuyen a una subafiliación significativa al Sistema General de Riesgos Laborales.

El presente trabajo tiene como objetivo revisar la literatura para identificar estudios publicados en bases de datos científicas que hayan sido desarrollados utilizando datos de SVE en el marco de la implementación de SG-SST o su equivalente en países de Latinoamérica. Se pretende evaluar si la información obtenida se utiliza como insumo para la toma de decisiones en salud pública. La pregunta de investigación planteada es: ¿qué información o datos provenientes de SVE, implementados a partir de un SG-SST u otro modelo de salud ocupacional según cada país, han sido utilizados en investigaciones publicadas en bases de datos científicas reconocidas en Latinoamérica?. Al abordar esta pregunta, se busca aportar conocimiento sobre la utilidad de los SVE en la mejora de las condiciones de salud laboral y su impacto en la formulación de políticas públicas que garanticen la seguridad y el bienestar de los trabajadores en la región. La identificación de estudios relevantes permitirá entender mejor cómo se utiliza la información epidemiológica en la toma de decisiones y en la gestión preventiva de la salud laboral, contribuyendo así a la protección y la promoción de la salud en el entorno de trabajo.

## METODOLOGÍA

Se llevó a cabo una revisión de la literatura, basada en artículos publicados en revistas científicas. Se definió un protocolo de búsqueda que incluyó la selección de descriptores pertinentes, asegurando su correspondencia entre los términos DeCS y MeSH relevantes para nuestra área de interés. También se establecieron palabras clave para complementar la búsqueda y se determinó la combinación de descriptores utilizando operadores booleanos (AND y OR). Se enumeraron las bases de datos por consultar y se especificaron las combinaciones de descriptores y palabras clave que debían emplearse en cada una de ellas. Se consideró la recolección de datos en una matriz que incluía variables como fecha de búsqueda, rango de antigüedad de los artículos, enlace para localizar cada artículo, disponibilidad del texto completo, referencia en formato Vancouver o APA, y país de origen.

### Criterios de elegibilidad

Se seleccionaron artículos que reportaran investigaciones epidemiológicas en el área de la salud ocupacional, realizadas en empresas o sectores económicos específicos y publicadas en revistas científicas indexadas entre 2010 y 2021 en Colombia o países de Latinoamérica. Se incluyeron estudios en inglés, español y portugués. Los artículos debían informar sobre el uso de datos provenientes de un SVE implementado en el marco de un SG-SST o su equivalente.

### Búsqueda de información

Se consultaron las principales bases de datos científicas en español e inglés, consideradas representativas: PubMed, ScienceDirect, Scielo, Scopus, Dialnet, Gale y Google Scholar. Los descriptores utilizados incluyeron términos DeCS: "Monitoreo Epidemiológico", "Salud Laboral", "Condiciones de Trabajo" y 'América Latina", y términos MeSH: "Epidemiological Monitoring", "Occupational Health", "Working Conditions" y "Latin America". Se combinaron estos términos considerando el número de resultados y la focalización del tema de interés. La búsqueda se limitó al monitoreo epidemiológico en el contexto de la salud laboral o las condiciones de trabajo de los empleados. Se hizo una búsqueda complementaria con las palabras clave: "vigilancia epidemiológica" y "salud ocupacional" en las bases de datos mencionadas y en Google Scholar, adaptando las estrategias de búsqueda según las herramientas disponibles en cada base de datos.

### Selección de los estudios

La revisión y selección la realizaron tres investigadores con estudios de especialización en el área de la salud ocupacional y la medicina laboral. Dado que la cantidad de publicaciones en el área principal fue escasa, se desestimó que los estudios incluidos contuvieran investigaciones basadas en datos derivados de un SVE formalmente establecido en las empresas o por empleadores, incluyendo para su revisión todos los estudios que se encontraran con la combinación de los descriptores propuestos y el periodo de tiempo fijado. Se eliminaron los estudios duplicados y se excluyeron aquellos elaborados en regiones del mundo distintas de Latinoamérica. Adicionalmente, se excluyeron los trabajos que contuvieran información de SVE no relacionados con el ambiente laboral o con la salud ocupacional.

### Proceso de extracción de datos

Se construyó una matriz para cada base de datos consultada, detallando los artículos encontrados según la combinación de descriptores y palabras clave, fecha de búsqueda, rango de antigüedad, formato de citación (APA o Vancouver) y disponibilidad del texto completo. Los artículos fueron revisados para verificar su cumplimiento con los criterios de elegibilidad conforme al objetivo y la pregunta de investigación. Los artículos se organizaron por fecha e idioma y se distribuyeron entre los tres investigadores para su revisión. Se evaluó la presencia de datos provenientes de SVE constituidos por empleadores o comités de SG-SST. Los estudios se categorizaron por temas, sectores económicos, fecha de publicación, país de origen, población estudiada y alcance.

### Riesgo de sesgo entre los estudios

Se interpretó cuidadosamente si los estudios utilizaban datos de SVE de un SG-SST implementado por empleadores. Esta claridad se correlacionó con los atributos de calidad de cada estudio. El sesgo potencial en la selección de artículos se relaciona con la combinación de descriptores. Sin embargo, al utilizar términos MeSH y DeCS exactos y realizar una búsqueda complementaria con palabras clave, se consideró que el riesgo de sesgo era mínimo, y se obtuvo una muestra aceptable.

### Medidas de resumen

Se calculó la frecuencia de estudios que usaron datos de SVE basados en SG-SST, desglosados por año de publicación, tipo de estudio, país u organización productora, y sector económico investigado. Se definió el lugar de la investigación, la institución o conjunto de instituciones involucradas, ya fueran hospitalarias, agrícolas, industriales, empresariales o áreas geográficas específicas. Se categorizó el tipo de afección de salud más frecuente en términos de daño, vulnerabilidad y riesgo.

### Análisis adicionales

Se evaluaron parámetros de calidad según el tipo de estudio, considerando la metodología de recolección de datos, el diseño del estudio, la pertinencia de las referencias, el riesgo de sesgo en la interpretación de los resultados y la consistencia interna. Se procuró obtener información consolidada que pudiera apoyar la necesidad y la relevancia de continuar promoviendo la existencia de datos provenientes de SVE en el ámbito de la salud laboral.

## RESULTADOS

De los artículos revisados, aproximadamente la mitad cumplían en gran medida con los criterios de selección. Algunos estudios ejemplifican la articulación entre el SVE y el SG-SST, aunque no surgieron con esa intención ni se proyectaron como políticas públicas generales. En estos estudios, el manejo de la información y la recolección de datos no se realizaba mediante un SVE, aunque en todos los casos se observaba una base fundamentada en el SG-SST 18. Esto sugiere que la unificación de información es una herramienta poderosa que solo será útil si se utiliza para la toma de decisiones y el desarrollo de políticas de salud relacionadas con el trabajo mediante la implementación de SVE en el contexto de SG-SST, como lo demuestran algunos estudios 13,19,20. Otros trabajos 21,22, aunque se centran en la vigilancia epidemiológica, adoptan un enfoque eminentemente clínico y consideran el tema en el marco de un sistema de gestión, lo que los aleja del SG-SST a pesar de su enfoque laboral. Un ejemplo notable es el trabajo de Toro-Osorio *et al.*^21^, que destaca la importancia de los resultados en términos de impacto sobre la salud pública debido a los riesgos de intoxicación con plaguicidas en zonas rurales. Sin embargo, este estudio no considera el SG-SST a pesar de sugerir intervenciones en áreas que son parte integral del sistema. El estudio de Comper y Padula 23 destaca la importancia de utilizar información en seguridad y salud en el trabajo para desarrollar políticas públicas que promuevan el control epidemiológico. Aunque no se enfoca específicamente en el SVE, subraya que las medidas deben implementarse a través de este programa para lograr una disminución en los indicadores de enfermedades laborales y accidentes de trabajo.

### Selección de estudios

**Figura 1:**
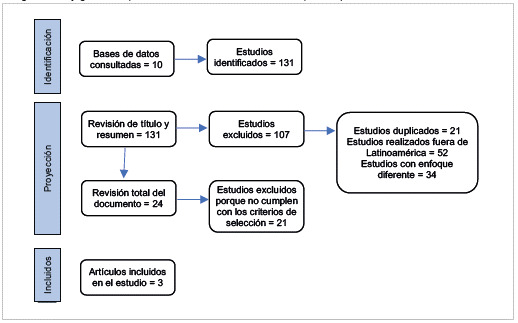
Flujograma del proceso de selección de los artículos que cumplieron con los criterios de inclusión

De acuerdo con la estrategia de búsqueda, se identificaron 131 artículos relacionados con los descriptores en las bases de datos seleccionadas, de los cuales se eligieron 24 para su examen detallado. Los 107 estudios restantes fueron excluidos por estar duplicados, realizarse fuera de Latinoamérica o tratar sobre vigilancia epidemiológica en temas ajenos a la salud laboral o las condiciones de trabajo. De los 24 artículos que cumplieron con los criterios de elegibilidad, 21 se consideraron hallazgos negativos porque, aunque mencionaban la vigilancia epidemiológica en el ámbito laboral, no utilizaban información proveniente de un SVE derivado de un SG-SST. Finalmente, solo tres documentos cumplían con las condiciones de elegibilidad de los SVE en el marco de la investigación propuesta (Figura 1).

### Distribución de estudios por países

**Figura 2:**
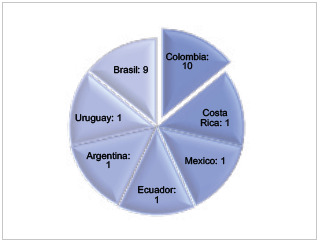
Distribución de los estudios realizados en Latinoamérica e incluidos para revisión

Los estudios se distribuyeron en países latinoamericanos: se encontró al menos un estudio en Colombia (10), Brasil (9), Uruguay (1), Costa Rica (1), México (1), Ecuador (1) y Argentina (1). Solo Colombia y Brasil demostraron tener estudios arraigados en el tema principal de esta investigación. En los demás países solo uno o ningún estudio aportaba evidencia científica relevante (Figura 2).

### Hallazgos en los principales sectores laborales

### Sector salud

En el sector salud se desarrollaron cinco estudios que abordan temas como la vigilancia de la exposición a radiaciones ionizantes 24 y la evaluación del impacto de las sobrecargas laborales en el estrés 20,25. Se encontraron iniciativas de monitoreo al personal de salud, exámenes ocupacionales, capacitaciones y foros para hacer seguimiento a las condiciones de salud de los trabajadores del área de enfermería y en el ambiente hospitalario 20,25,26. Un estudio abarcó la temática de las afectaciones de tipo psicosocial 19.

### Sector minero, industrial y de hidrocarburos

Cuatro estudios abordaron este sector, investigando la afectación de la salud pulmonar por exposición al polvo en minas de carbón 27, afecciones osteomusculares en operadores de cadenas manufactureras 28, obreros mineros 27 y la hematotoxicidad y el riesgo de carcinogénesis en trabajadores expuestos a hidrocarburos 29.

### Sector agroindustrial

En el sector agrícola se encontraron estudios sobre la exposición a tóxicos. Por ejemplo, un estudio detectó trastornos musculoesqueléticos en mujeres recolectoras de mariscos en Bahía, Brasil 30. Otro estudio trató la exposición a organofosforados y carbamatos en jornaleros y trabajadores del campo en 11 departamentos de Colombia 18. Un estudio reciente cuantificó niveles de colinesterasa en caficultores del departamento de Caldas expuestos a plaguicidas 21. Otros trabajos se centraron en embolsadores de fincas bananeras en Urabá con exposición a agrotóxicos 31 y agricultores de cultivos de arroz en el Tolima expuestos a plaguicidas (32), utilizando metodologías cualitativas para explorar los determinantes sociales de las intoxicaciones.

#### Sector ocupacional

Se llevaron a cabo varias intervenciones para evaluar la implementación de los SG-SST. Un estudio transversal investigó el nivel de implementación de programas de SG-SST en 73 empresas de Antioquia 33. En Centro-américa se llevó a cabo una encuesta sobre condiciones de trabajo y salud en seis países: Costa Rica, El Salvador, Guatemala, Honduras, Nicaragua y Panamá, enfocada en afecciones musculoesqueléticas, debido a la falta de datos generales sobre este tema en la región 34. También se desarrollaron indicadores para la vigilancia de la salud ocupacional en América Latina y el Caribe mediante un informe especial basado en un consenso de expertos de Ecuador y Argentina 35. En Brasil, una revisión sistemática identificó 215 artículos sobre encuestas ocupacionales, que abarcaban temas como problemas mentales, musculoesqueléticos, vocales, auditivos, accidentes laborales, exposiciones ambientales y hábitos de vida 28. A pesar del número de artículos revisados, pocos se enfocaron en la vigilancia de la salud ocupacional.

### Estudios objeto de la revisión

Solo tres investigaciones utilizaron información proveniente de un SVE derivado de un SG-SST. De estos, dos presentaron análisis transversales, mientras que uno fue un estudio retrospectivo longitudinal realizado en un hospital universitario en Uruguay, vigilando la exposición a radiaciones ionizantes (36). Es ideal que estos SVE existan en áreas de alto riesgo de carcinogénesis, como los servicios de radiología hospitalarios. Los otros dos estudios descriptivos transversales se desarrollaron en el sector industrial, implementando un SVE para detectar desórdenes osteomusculares en trabajadores de una empresa de refrigeradores en Barranquilla, Colombia 28, y en la mina del Cerrejón en la Guajira, Colombia, para prevenir desórdenes musculoesqueléticos 27 (Tabla 1).

**Tabla 1:** Análisis de los estudios que cumplieron con los criterios de elegibilidad

Referencia	Objetivo	Método	Conclusión
19	Analizar los factores psicosociales tanto intralaborales como extralaborales que afectan a los empleados de una institución del sector salud que ofrece servicios de alta complejidad (nivel III).	Análisis cuantitativo y de tipo transaccional descriptivo. Se realizó un muestreo aleatorio simple y un muestreo aleatorio estratificado con asignación proporcional por área de trabajo.	En conclusión, la clínica presenta un alto nivel de riesgo psicosocial, lo que hace necesaria la implementación de un Programa de Vigilancia Epidemiológica en Riesgo Psicosocial para prevenir el estrés y las enfermedades relacionadas.
27	Evaluar los diferentes dominios de la ergonomía ha implementado Cerrejón en los últimos años.	Estudios analíticos para conocer prácticas operacionales en el proceso de cargue del camión, los niveles de aceleración diaria entre operadores de equipo minero y las medidas antropométricas en comunidades indígenas.	La elevada frecuencia de falsas alarmas y ruido enmascara las señales auditivas del sistema anticolisión y del CTD, lo que causa molestia a los conductores y afecta negativamente su capacidad de respuesta y atención.
36	Exponer los hallazgos del programa de monitoreo en salud ocupacional para los trabajadores universitarios que estuvieron en contacto con radiaciones ionizantes en el período 2003-2006.	Estudio descriptivo retrospectivo longitudinal. La valoración de la exposición se realizó mediante dosimetría de filme.	El monitoreo de la exposición ha facilitado la dirección del control médico periódico específico y la intensificación de las acciones de radioprotección. En esta línea, el departamento de Salud Ocupacional está llevando a cabo tareas de educación y difusión del programa para fortalecer las medidas de prevención.

#### Controversias entre estudios

En tres estudios hubo controversias sobre el uso de datos de un SVE en SG-SST. Realizados por autores brasileños a partir de la misma fuente de información, el Sistema de Vigilancia de la Salud de los Trabajadores de Enfermería 20,25,26, este *software* obtenía datos estadísticos sobre cargas de trabajo y perfiles patológicos. Sin embargo, no quedó claro si esta estrategia formaba parte de un SVE sistemático implementado por empleadores, era una iniciativa derivada de la investigación de los autores, o resultaba de la integración vertical del sistema de salud regional.

En conclusión, aunque solo una minoría de los estudios revisados cumplieron estrictamente con los criterios de utilizar un SVE derivado de un SG-SST, aquellos que lo hicieron evidencian la relevancia de estos sistemas para la vigilancia y la mejora de la salud laboral. Los hallazgos sugieren que la integración de SVE en el SG-SST puede ser crucial para desarrollar políticas de salud ocupacional efectivas y reducir enfermedades laborales y accidentes.

## DISCUSIÓN

La revisión de la literatura y los estudios evaluados revelan una preocupante falta de programas de vigilancia epidemiológica (SVE) en varios sectores laborales de Latinoamérica. En sectores críticos como la industria alimentaria, hay una notable tendencia al subdiagnóstico de enfermedades laborales debido al desconocimiento de los factores de riesgo por parte de los profesionales de la salud y las autoridades pertinentes 37,38. Este subdiagnóstico conduce a que muchas enfermedades se clasifiquen erróneamente como de origen común, lo que resulta en una falta de seguimiento adecuado y estadísticas precisas, contribuyendo al aumento de la morbilidad y la mortalidad relacionadas con el trabajo.

A pesar de que la normativa proporciona definiciones claras sobre las condiciones laborales, la evaluación de riesgos y las obligaciones de empleadores y trabajadores, en la práctica, la aplicación de estas normas es deficiente. Las pequeñas y medianas empresas (pymes), que representan una parte significativa de la economía, enfrentan desafíos particulares para cumplir con los estándares mínimos exigidos, como la afiliación de los trabajadores al sistema de seguridad social integral y la implementación de medidas de prevención y control; además, la tabla de enfermedades laborales en Colombia prioriza solo un pequeño segmento de enfermedades, ignorando muchas condiciones que afectan la salud de los trabajadores 13. Una de las soluciones propuestas es la creación de sistemas de información integrados que enlacen la historia médica y laboral de los trabajadores. Esto podría garantizar una cobertura más completa y el cumplimiento de los derechos de salud, especialmente para los trabajadores independientes e informales, quienes a menudo están excluidos de los sistemas de seguridad social 39. Estudios previos, como el de Rojas y Vecino 40, han señalado las dificultades de articular distintas fuentes de información, como registros de cáncer, mortalidad y exposiciones ocupacionales, para evaluar adecuadamente la fracción de cáncer atribuible a la ocupación. Estos desafíos son comunes incluso en países desarrollados, lo que sugiere que la situación en Latinoamérica es aún más crítica. Investigaciones en España han mostrado que una gran parte de las enfermedades laborales no son reconocidas como tales, lo que afecta especialmente a los casos graves que deberían recibir prestaciones correspondientes 41. Este problema de subnotificación refleja una falta de reconocimiento de la verdadera incidencia y prevalencia de las enfermedades laborales y resalta la necesidad de mejorar los sistemas de vigilancia y reporte.

La revisión de los artículos también revela que la mayoría de ellos destacan la necesidad de SVE consolidados en el ámbito laboral. Por ejemplo, Varona *et al.*^32^ encontraron que los factores culturales pueden ser un obstáculo significativo para la salud laboral, como en el caso de la exposición a plaguicidas en los agricultores de arroz en Colombia, donde el uso de equipos de protección personal (EPP) puede ser mal visto. En otro ejemplo, Aguirre-Buitrago *et al.*^31^ señalaron que, a pesar de la integración vertical con multinacionales, la supervisión del uso de agrotóxicos en las fincas bananeras es insuficiente, y la vigilancia básica existente no genera datos útiles. En el sector salud, Oliveira y Andres (26) imple-mentaron un sistema de monitoreo basado en *software* para enfermeros en hospitales universitarios en Sao Paulo, pero encontraron limitaciones significativas en la recolección de datos, y únicamente tuvieron acceso a información sobre la frecuencia de accidentes laborales y licencias por enfermedad. Aunque estas aproximaciones muestran la relación entre el nivel de riesgo y la frecuencia de eventos adversos en salud, es necesario desarrollar metodologías y estructuras más completas para sistematizar una mayor cantidad de información útil. Otro estudio utilizó múltiples fuentes de datos, como informes gerenciales y registros de turnos de trabajo, para complementar la información primaria sobre accidentes laborales 25. Sin embargo, esta actividad aún no está sistematizada en las instituciones hospitalarias, lo que destaca la necesidad de integrar estos procesos en la gestión diaria de la salud ocupacional.

En conclusión, a pesar de la normativa que obliga a las empresas en países como Colombia a tener un SG-SST del cual debe derivarse un SVE, las investigaciones de la última década muestran una ausencia significativa de estos sistemas y una falta de cultura en SG-SST y promoción de la salud. La generación de datos sistemáticos, confiables y comparables es esencial para retroalimentar las políticas públicas que garanticen la seguridad social y la salud de los trabajadores. Urge la implementación generalizada de SVE en el ámbito laboral para comprender la verdadera prevalencia e incidencia de enfermedades y accidentes laborales. Esto permitiría identificar condiciones de salud y patologías que han sido pasadas por alto y reconocer factores de riesgo que puedan ser intervenidos para fomentar una cultura de prevención. La mayoría de los estudios revisados son iniciativas académicas que señalan la ausencia de generación sistemática de datos por parte de los empleadores. La implementación de SVE sólidos y la creación de una cultura de prevención no solo mejorarán el bienestar de los trabajadores, sino que proporcionarán una base sólida para desarrollar políticas públicas efectivas. En resumen, para garantizar la salud y la seguridad de los trabajadores, es imperativo que los empleadores adopten prácticas de vigilancia epidemiológica adecuadas y que los sistemas de salud laboral sean fortalecidos y sistematizados. La creación de SVE integrados y la promoción de una cultura de prevención son pasos cruciales para lograr un ambiente laboral más seguro y saludable en Latinoamérica ♦
